# Wnt signaling-mediated activation of cancer cell stemness in thoracic SMARCA4-deficient undifferentiated tumor cells

**DOI:** 10.1038/s41420-026-03224-6

**Published:** 2026-06-30

**Authors:** Yan Xu, Lumei Wang, Hongjia Zhang, Lin Liang, Hui Han, Tianyu Xiao, Cheng Zhang, Qiye He, Zuoren Yu, Jinli Gao

**Affiliations:** 1 Department of Pathology, Shanghai East Hospital, Tongji University School of Medicine, Shanghai, 200123 China; 2 Research Center for Translational Medicine, Shanghai East Hospital, Tongji University School of Medicine, Shanghai, China; 3 Sino-German East Virchow Joint Pathology Research Institute, Shanghai East Hospital, Tongji University School of Medicine, Shanghai, China

**Keywords:** Oncogenesis, Non-small-cell lung cancer

## Abstract

Thoracic SMARCA4-deficient undifferentiated tumors (SMARCA4-UT) represent a distinct and highly aggressive malignancy characterized by SMARCA4 deficiency, poor prognosis, and resistance to chemotherapy. The lack of clarity regarding the regulatory mechanisms underlying this disease has hindered the development of standardized treatment guidelines. In this study, we analyzed clinical samples and found significantly elevated levels of cancer stem cell markers, including SOX2, CD34, c-Myc, and EpCam, in thoracic SMARCA4-UT, which were further confirmed in SMARCA4-deficient non-small cell lung cancer (NSCLC) cells. Furthermore, SMARCA4 knockdown in NSCLC cells promoted cell proliferation, migration, invasion, and tumorsphere formation in vitro, as well as enhanced tumor growth in a mouse model in vivo. RNA sequencing analysis of SMARCA4-deficient cells revealed the upregulation of a subset of genes, including Wnt10A, and activation of Wnt signaling. This was validated through SMARCA4-ChIP-seq and SMARCA4-ChIP-PCR analyses, Wnt activity reporter assays, and subcellular distribution analysis of β-catenin. Subsequent studies using a small molecule Wnt inhibitor in SMARCA4-deficient cells and thoracic SMARCA4-UT patient-derived organoids demonstrated induction of apoptosis, inhibition of cell stemness, and suppression of tumor cell growth. These results suggest the potential for developing a novel therapeutic strategy targeting cancer stem cells for the treatment of thoracic SMARCA4-UT.

## Introduction

Thoracic SMARCA4-deficient undifferentiated tumor (SMARCA4-UT) is a rare and highly aggressive malignancy with a poor overall prognosis. It is recognized as a distinct entity in the Fifth Edition of the WHO classification, owing to its unique histology, immunohistochemistry, clinical features, and prognostic outcomes. SMARCA4-UT is characterized by an undifferentiated rhabdoid morphology and SMARCA4 deficiency. SMARCA4 gene encodes the Brahma-related gene 1 (BRG1) protein, serving as a catalytic core subunit of the switch/sucrose nonfermenting (SWI/SNF) chromatin remodeling complex [1–3]. This tumor type primarily affects young to middle-aged adults, and presents as a large mass that compresses the tissues surrounding the mediastinum and lung parenchyma. SMARCA4-UT has a high recurrence rate after surgical resection, is resistant to radiotherapy, and shows poor responses to chemotherapy, with a median overall survival of only 4–7 months [4]. As a result, thoracic SMARCA4-UT poses a significant clinical challenge that urgently needs to be addressed.

Cancer stem cells (CSCs), also referred to as tumor-initiating cells (TICs), exhibit stem cell-like properties, including self-renewal, differentiation capacity, tumor development, metastasis, and resistance to therapy. Several CSC markers have been identified in non-small cell lung cancer (NSCLC), including SOX2, c-Myc, and CD34. Previous studies have shown that thoracic SMARCA4-deficient tumors are positive for CD34 and SOX2 [5, 6]. Notably, SMARCA4 and SOX2 play critical roles in the diagnostic evaluation of SMARCA4-UT. The characteristics of SMARCA4-UT cells, such as high invasiveness, strong tumorigenicity, immune evasion, and treatment resistance, are also hallmarks of cancer stem cells.

Various signaling pathways, including Wnt/β-catenin, PI3K/AKT, and Notch, have been implicated in regulating CSCs [7]. The Wnt family proteins have been well demonstrated to regulate stem cell self-renewal and progenitor cell proliferation/differentiation through canonical or non-canonical pathways, contributing to the development and progression of human cancers, including NSCLC [8]. However, the role of the Wnt pathway in regulating cancer cell stemness in thoracic SMARCA4-UT remains unclear.

Herein, we utilized clinical patient specimens, SMARCA4-deficient NSCLC cells, tumor-bearing animal models, and patient-derived organoids to determine the cancer-related phenotypes and differentially expressed genes caused by SMARCA4 deficiency. As a result, we observed stemness acquisition and overexpression of CSC markers, including SOX2, CD34, and c-Myc, in SMARCA4-deficient cancer cells, as well as upregulation of Wnt10A and activation of Wnt signaling. The combination of a small-molecule Wnt signaling inhibitor and chemotherapy exhibited a synergistic therapeutic effect on both SMARCA4-deficient tumor-bearing mice and thoracic SMARCA4-UT patient-derived organoids. The current study not only reveals a pathological mechanism mediating the development and progression of thoracic SMARCA4-UT, but also provides new therapeutic strategies for patients with this disease.

## Results

### Loss of SMARCA4 and enhanced level of Ki67 in thoracic SMARCA4-UT clinical samples

**Fig. 1:**
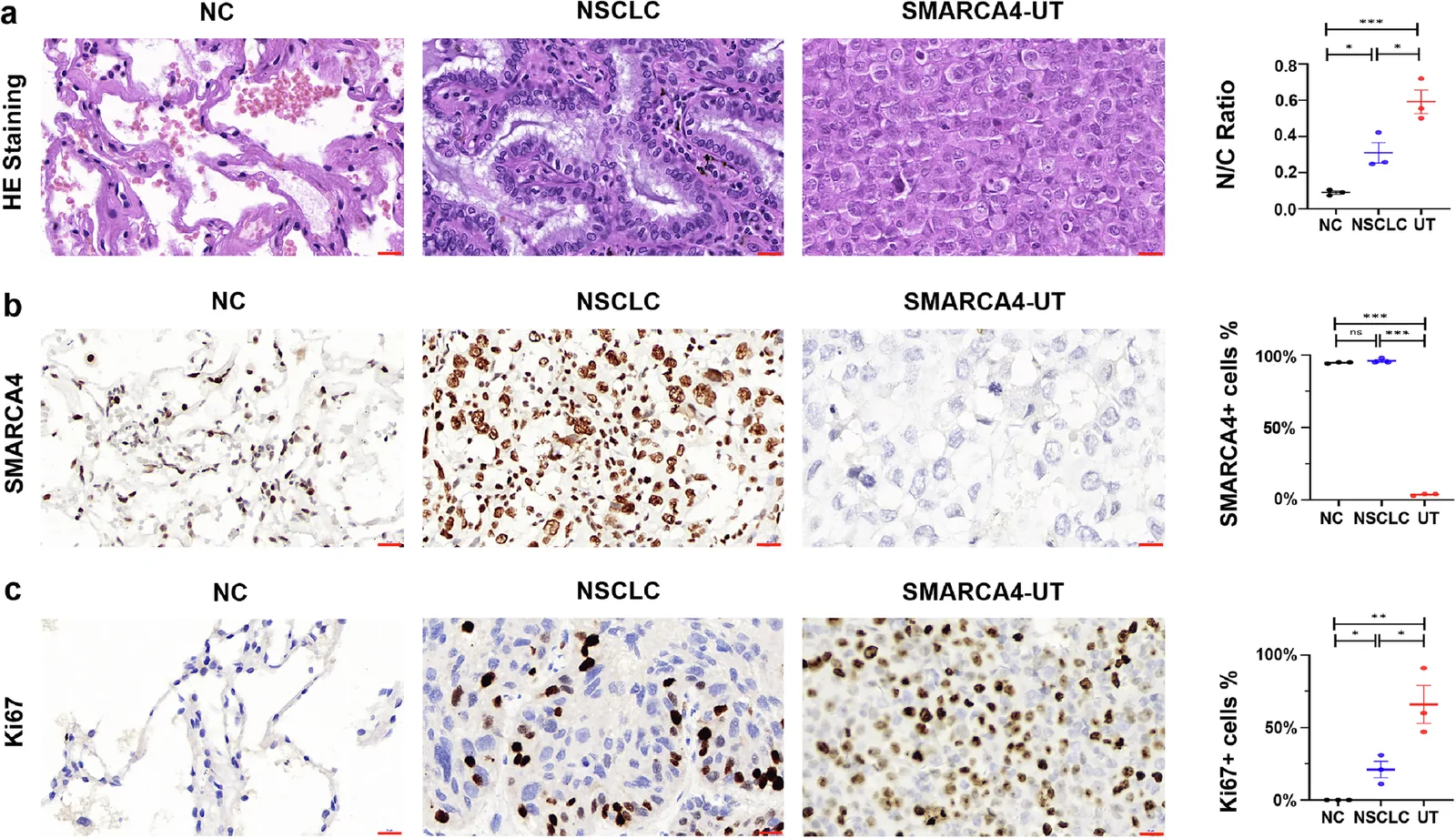
Loss of SMARCA4 and enhanced level of Ki67 in thoracic SMARCA4-UT clinical samples. Hematoxylin-eosin (HE) staining (**a**), immunohistochemistry (IHC) staining of SMARCA4 (**b**) and Ki67 (**c**) in clinical samples of thoracic SMARCA4-UT, non-small cell lung cancer (NSCLC), and normal controls (NC). N/C ratio: nuclear-to-cytoplasmic (N/C) ratio. Scale bar: 20 µm. *P ≤ 0.05; **P ≤ 0.01; ***P ≤ 0.001. *n* = 3 in each group.

We compared pathological features between clinical samples from patients with non-small cell lung cancer (NSCLC), thoracic SMARCA4-UT, and normal control (NC) lung tissues. As shown in Fig.1a, a much higher nuclear-to-cytoplasmic (N/C) ratio was observed in SMARCA4-UT tissues, compared to NSCLC or NC. In addition, only 3.5% of SMARCA4-positive cells were found in SMARCA4-UT, compared to ~95% in either NC or NSCLC (Fig.1b). We further analyzed the levels of Ki67, a marker of cell proliferation, and found upregulation of Ki67 in both SMARCA4-UT and NSCLC, compared to NC (Fig.1c). Notably, ~66% and 21% of Ki67-positive cells were found in SMARCA4-UT and NSCLC, respectively (Fig.1c).

### Overexpression of CSC markers in thoracic SMARCA4-UT clinical samples

**Fig. 2:**
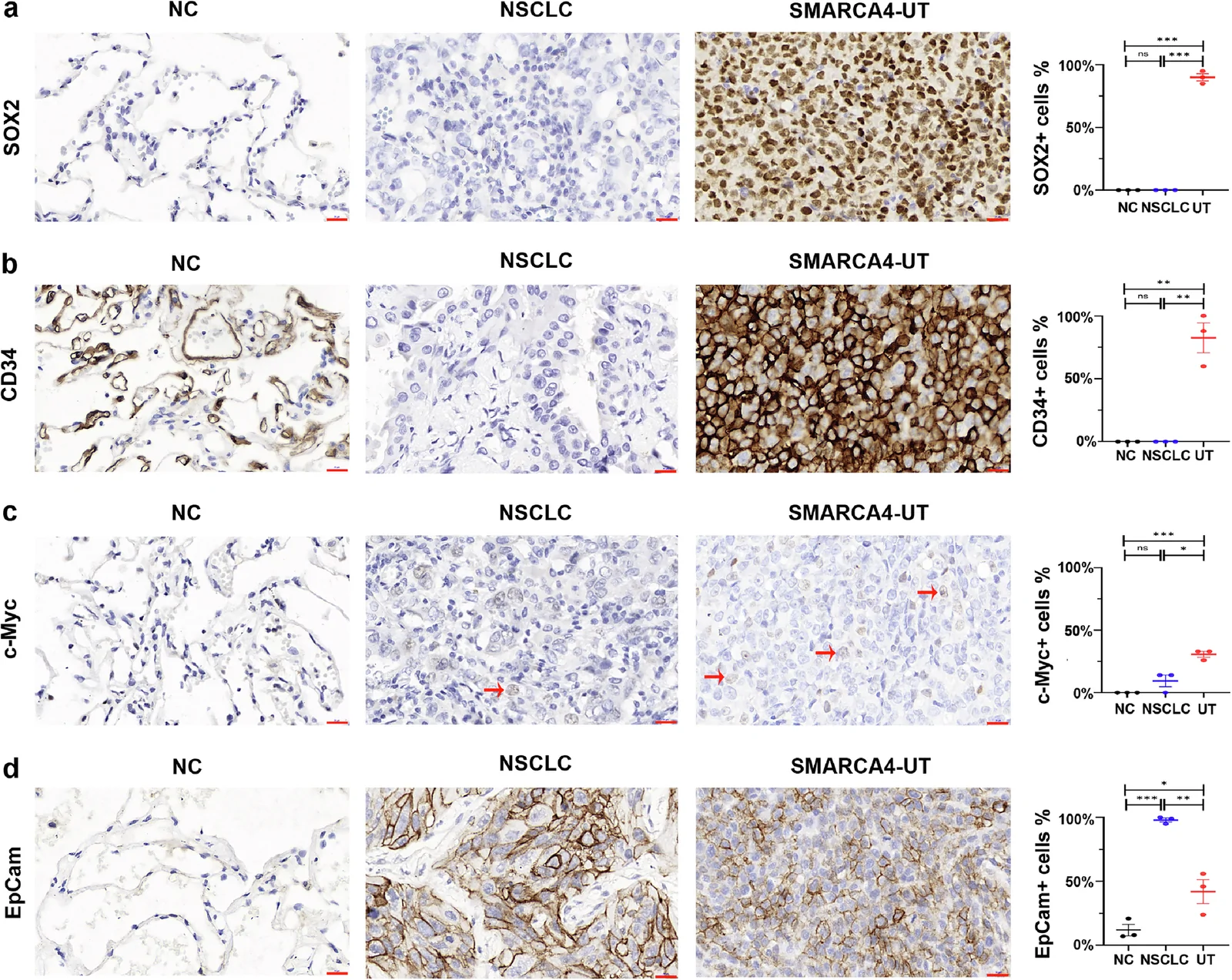
Cancer cell stemness analysis in clinical samples. Immunohistochemistry (IHC) staining of SOX2 (**a**), CD34 (**b**), c-Myc (**c**), and EpCam (**d**) in clinical samples of thoracic SMARCA4-UT, NSCLC, and normal controls (NC). Scale bar: 20 µm. ns: no significance; *P ≤ 0.05; **P ≤ 0.01; ***P ≤ 0.001.

To investigate the relationship between SMARCA4 deficiency and stemness acquisition in cancer cells, three CSC markers including SOX2, CD34, c-Myc, and EpCam were analyzed in the three types of tissue slides (NSCLC, SMARCA4-UT, and NC; Fig. 2a–d). The immunohistochemistry (IHC) staining analysis revealed that 90% and 82.7% of cells in the SMARCA4-UT tissues were positive for SOX2 and CD34 immunoreactivity, respectively, while their expression levels were very low to even undetectable in both NC and NSCLC tissues (Fig. 2a, b). c-Myc is one of the core markers for pluripotent stem cells [9], playing critical roles in CSCs [10, 11]. Our IHC staining analysis showed that c-Myc-positive cell percentage was much higher in SMARCA4-UT ( ~ 30.7%) than that in NSCLC ( ~ 9.3%), while a very low expression in NC (Fig. 2c). EpCam, a transmembrane glycoprotein, has shown aberrant overexpression in multiple types of carcinomas [12, 13], and is associated with CSCs [14]. Our IHC analysis showed that EpCam was significantly upregulated in both SMARCA4-UT and NSCLC, compared to a very low level in NC (Fig. 2d).

### SMARCA4 deficiency promoted cell proliferation, migration, invasion, and stemness in lung cancer cells

**Fig. 3:**
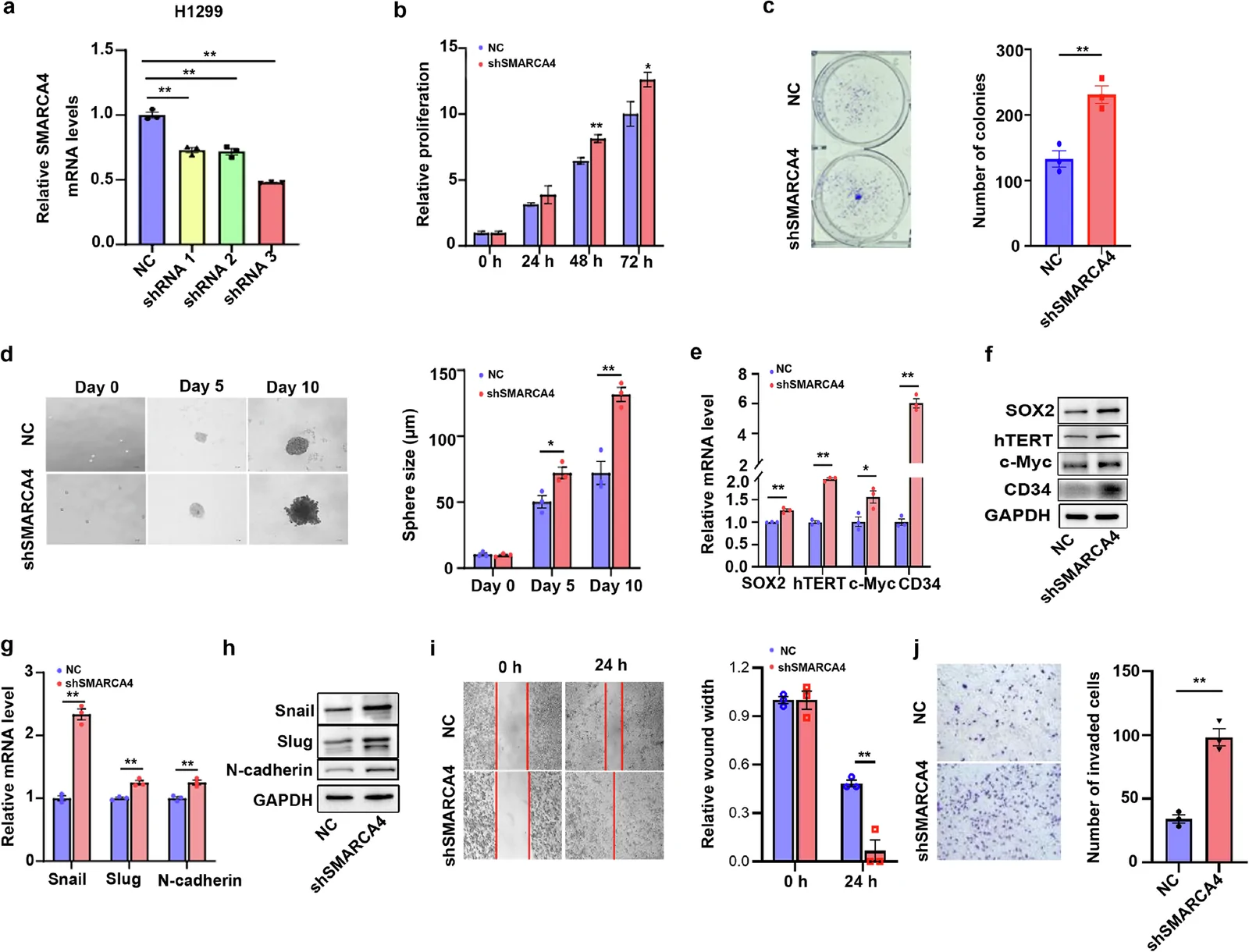
SMARCA4 deficiency promoted cell proliferation, migration, invasion, and stemness in H1299 cancer cells. **a** SMARCA4 mRNA levels in H1299 cells were significantly reduced by shRNAs targeting SMARCA4, with shRNA3 selected for further analysis. Cell proliferation analysis in H1299 cells with or without knockdown of SMARCA4 using the CCK-8 assay (**b**) and the colony formation assay (**c**). Analysis of cancer cell stemness in H1299 cells with and without SMARCA4 knockdown using the tumorsphere formation assay (**d**) and gene expression examination of cancer stem cell markers SOX2, hTERT, c-Myc and CD34 (**e**, **f**). Gene expression analysis of epithelial-mesenchymal transition (EMT) markers Snail, Slug and N-cadherin (**g**, **h**), as well as cell migration (**i**) and invasion (**j**) analyses in H1299 cells with and without SMARCA4 knockdown. *P ≤ 0.05; **P ≤ 0.01.

In view of the increased aggressiveness and stronger stemness due to loss of SMARCA4 in thoracic SMARCA4-UT cells, we investigated phenotypes induced by SMARCA4 deficiency in lung cancer cells. SMARCA4 was knocked down in an NSCLC cell line H1299 by applying lentiviral-based shRNAs targeting SMARCA4 (Fig. 3a), followed by a series of cancer-related functional assays.

As shown in Fig. 3b of CCK-8 assay and Fig. 3c of colony formation assay, knockdown of SMARCA4 in H1299 cells significantly promoted cell proliferation, compared to vector control. Next, we conducted an in vitro tumorsphere formation assay to investigate the effect of SMARCA4 deficiency on cancer cell stemness. As a result, spheres formed from SMARCA4 knockdown cells showed a significantly larger size than that from control cells (Fig. 3d, ~125 μm vs ~63 μm in diameter at day 10.). We further examined the expression of CSC markers including SOX2, CD34, c-Myc and hTERT in H1299 cells with or without knockdown of SMARCA4. As shown in Fig. 3e, f, all four CSC marker genes showed upregulation at both mRNA and protein levels after SMARCA4 knockdown.

In view of the close correlation between CSCs and cancer metastasis, we additionally investigated epithelial-mesenchymal transition (EMT) in H1299 cells with or without knockdown of SMARCA4, as well as cell migration and invasion. As shown in Fig. 3g, h, the expression of three EMT markers including Snail, Slug, and N-cadherin was significantly induced in SMARCA4 knockdown cells, which were validated at both mRNA and protein levels. Consistently, wound-healing assays demonstrated a significant increase of cell migration in SMARCA4 knockdown cancer cells (Fig. 3i). And transwell assays demonstrated a significant increase of cell invasion after SMARCA4 knockdown (Fig. 3j).

**Fig. 4:**
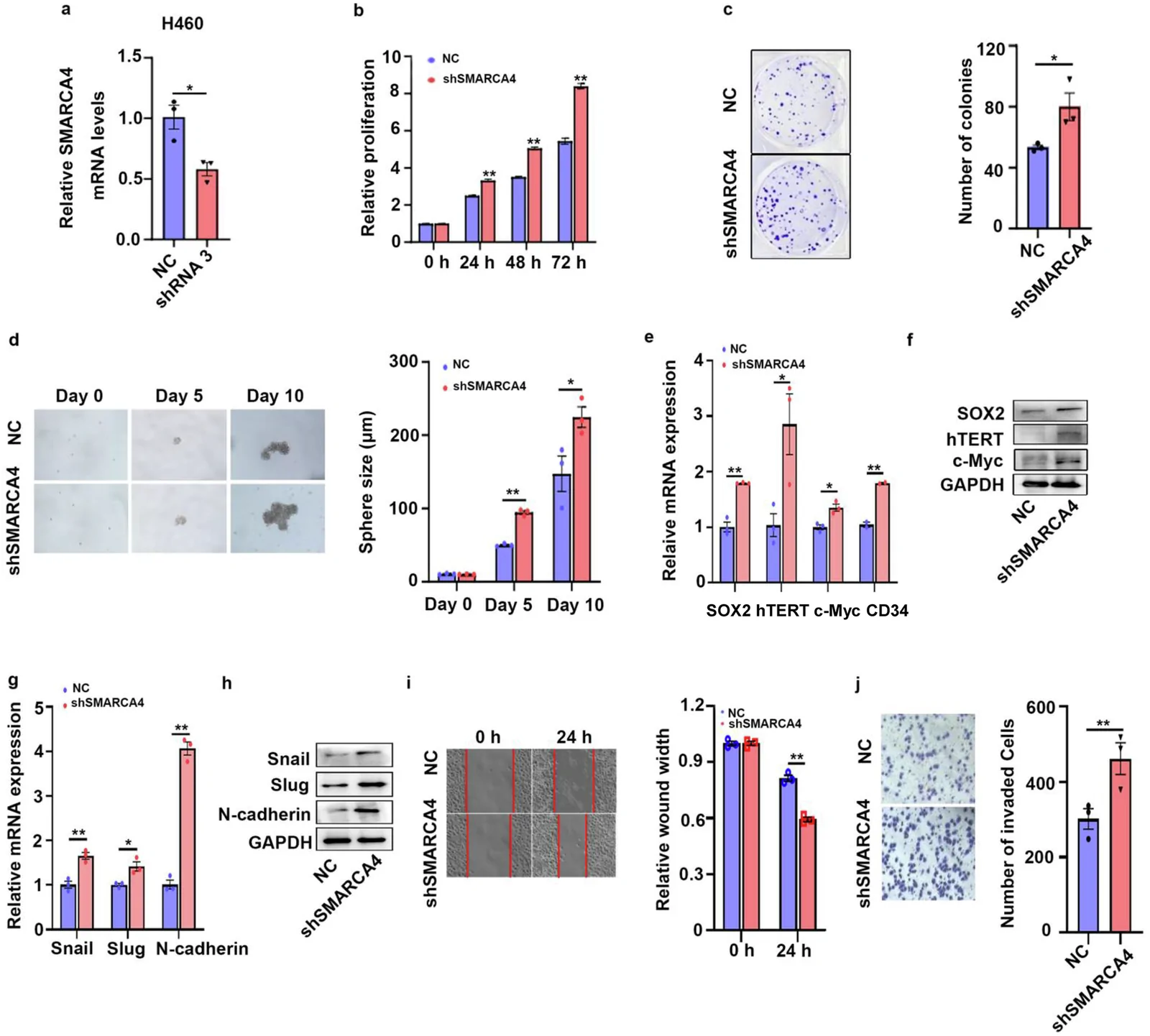
SMARCA4 deficiency promoted cell proliferation, migration, invasion, and stemness in H460 cancer cells. **a** SMARCA4 mRNA levels in H460 cells were significantly reduced by shRNA3 targeting SMARCA4. Cell proliferation analysis in H460 cells with and without knockdown of SMARCA4 using the CCK-8 assay (**b**) and the colony formation assay (**c**). Analysis of cancer cell stemness in H460 cells with or without SMARCA4 knockdown using the tumorsphere formation assay (**d**) and gene expression examination of cancer stem cell markers SOX2, hTERT, c-Myc and CD34 (**e**, **f**). Gene expression analysis of epithelial-mesenchymal transition (EMT) markers Snail, Slug, and N-cadherin (**g**, **h**), as well as cell migration (**i**) and invasion (**j**) analyses in H460 cells with or without SMARCA4 knockdown. *P ≤ 0.05; **P ≤ 0.01.

To further confirm the oncogenic induction caused by SMARCA4 deficiency, another NSCLC cell line, H460, was infected with a shRNA targeting SMARCA4. Subsequently, we analyzed cell proliferation, migration, invasion, and CSC properties. As shown in Fig. 4a– j, we observed results in H460 cells that were consistent with those obtained in H1299 cells (Fig. 3), confirming that SMARCA4 knockdown in lung cancer cells can induce increased aggressiveness and stemness, similar to thoracic SMARCA4-UT.

### SMARCA4 deficiency promoted tumor growth in vivo

**Fig. 5:**
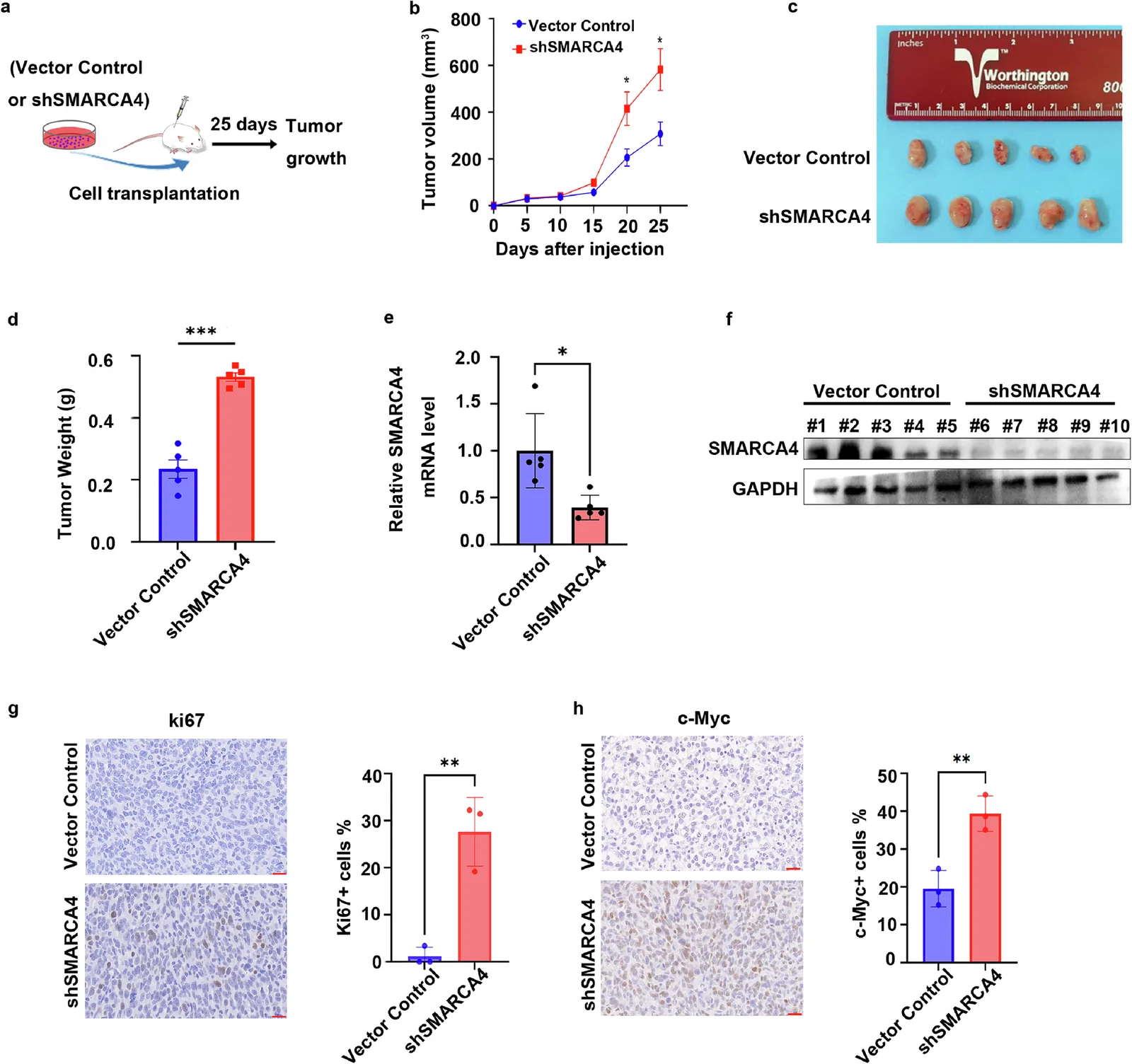
SMARCA4-deficiency promoted tumor growth in vivo. **a** A schematic representation of the H1299 cell transplantation-derived tumor mouse study. **b** Tumor growth curves indicating that tumors derived from SMARCA4-deficient cells grew significantly faster than those in the control group. Tumor images (**c**) and tumor weights (**d**) from each mouse in the SMARCA4-deficient group, compared to the control group. Confirmation of SMARCA4 expression in tumors from both SMARCA4-deficient and control mice, conducted *via* western blot (**f**) and quantitative RT-PCR (**e**). IHC analysis of Ki67 (**g**) and c-Myc (**h**) in SMARCA4-deficient tumors, compared to controls. *P ≤ 0.05; **P ≤ 0.01; ***P ≤ 0.001.

In order to assess the effects of SMARCA4 deficiency on tumor growth in vivo, H1299 cells treated with either shRNA-SMARCA4 or a negative control were transplanted into nude mice *via* subcutaneous injection (*n* = 5 per group). Tumor growth was monitored for 25 days (Fig. 5a). The tumor growth curves clearly demonstrated that tumors derived from SMARCA4-deficient cells grew significantly faster than those in the control group (Fig. 5b). On day 25 after cell transplantation, the mice were euthanized, and the tumors were excised for further analysis (Fig. 5c). Consistent with the results shown in Fig. 5b, tumors in the SMARCA4-deficient group were heavier than those in the control group (Fig. 5d).

Further analyses were conducted on these tumors including the examination of expression levels of SMARCA4, Ki67, and c-Myc. As indicated in Fig. 5e, f, the knockdown of SMARCA4 by shRNA treatment was confirmed at both mRNA and protein levels in these tumors. IHC analysis revealed higher expression levels of both Ki67 and c-Myc in the SMARCA4-deficient tumors, compared to controls (Fig. 5g, h).

### Upregulation of Wnt10A and activation of Wnt/β-catenin signaling in the SMARCA4-deficient cancer cells

**Fig. 6:**
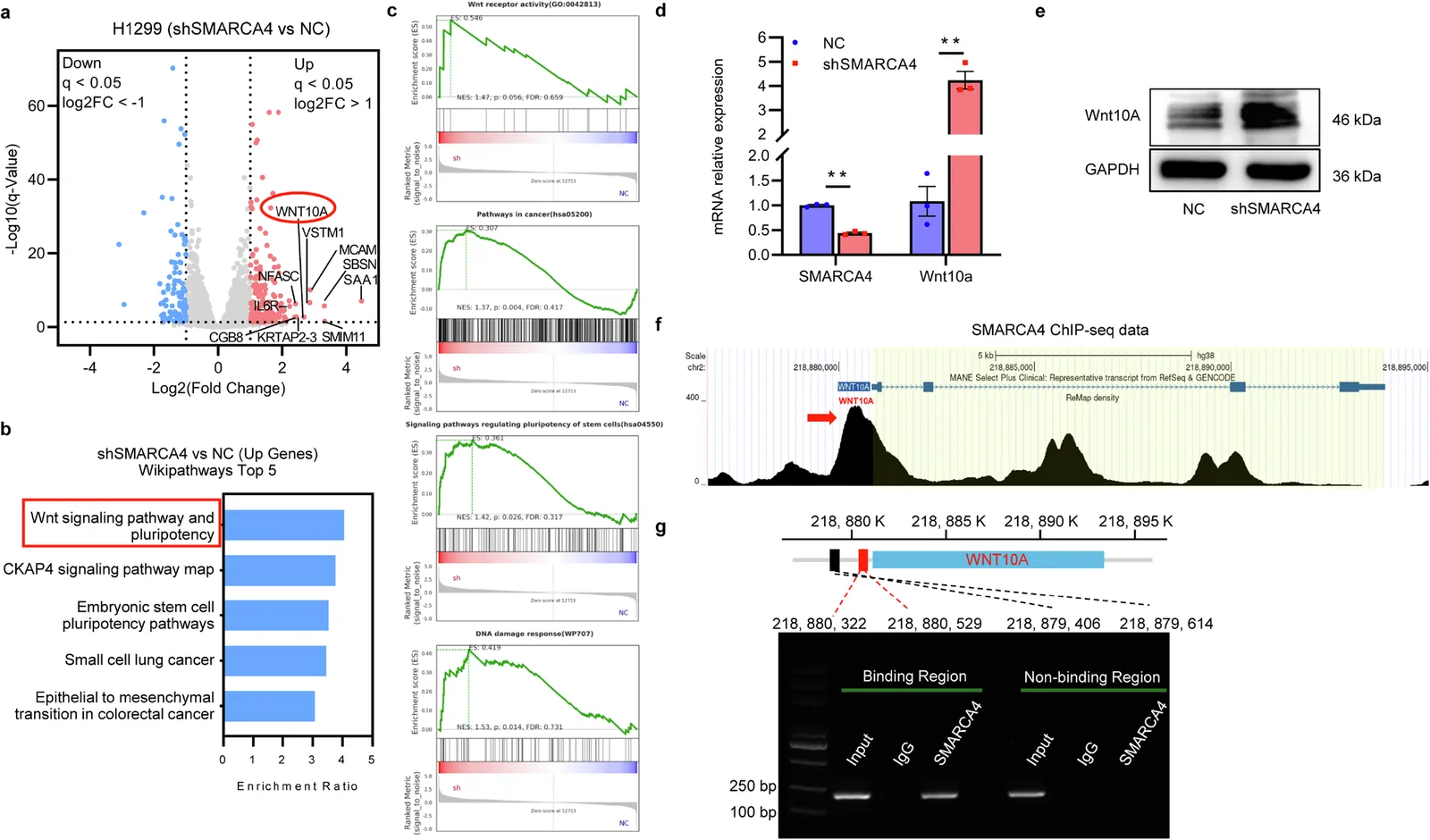
Upregulation of Wnt10A in SMARCA4-deficient cancer cells. **a** Volcano plot showing the differentially expressed genes (DEGs) regulated by SMARCA4 in H1299 cells. The top 10 upregulated genes are indicated, including Wnt10A. **b** The top five most enriched signaling pathways identified through WikiPathway enrichment analysis of the upregulated DEGs. **c** Gene Set Enrichment Analysis (GSEA) indicating enrichment of pathways including Wnt signaling, cancer regulation, stem cell regulation and DNA damage response. RT-qPCR (**d**) and western blot (**e**) analysis confirming upregulation of Wnt10A in SMARCA4-deficient H1299 cells. **f** ChIP-seq analysis showing a strong SMARCA4 binding peak (solid arrow in red) at the Wnt10A promoter region in H1299 cells. **g** SMARCA4 ChIP-PCR analysis confirming the amplification of the Wnt10A promoter in DNA pulled down by SMARCA4 ChIP in H1299 cells.

In order to determine the mechanisms underlying tumorigenesis induced by SMARCA4 deficiency, we conducted an RNA-seq analysis on H1299 cells treated with either shRNA-SMARCA4 or a negative control. Differentially expressed genes (DEGs) were identified using a cutoff of fold change ≥ 2 and an adjusted *p* value of <0.05. As a result, we found that 263 genes were upregulated while 141 genes were downregulated in SMARCA4-deficient H1299 cells (Fig. 6a). WikiPathway enrichment analysis of the upregulated DEGs revealed the top five most enriched signaling pathways, including Wnt signaling, CKAP4 signaling, small cell lung cancer, embryonic stem cell pluripotency, and epithelial-mesenchymal transition (EMT) (Fig. 6b). GSEA analysis of the gene sets differentially expressed in SMARCA4 knockdown (shSMARCA4) H1299 cells indicated enrichment of pathways including Wnt signaling, cancer regulation, stem cell regulation, DNA damage response, etc. (Fig. 6c).

Notably, Wnt10A, as a key member of the Wnt ligand family, is one of the top 10 upregulated genes in the RNA-seq data above. The upregulation of Wnt10A in SMARCA4-deficient H1299 cells was confirmed at both mRNA and protein levels (Fig. 6d, e). In order to investigate the mechanism by which SMARCA4 regulates Wnt10A expression, we analyzed human SMARCA4 ChIP-seq data from the ChIP-Atlas database [15] and identified a strong SMARCA4 binding peak at the Wnt10A promoter region (Fig. 6f). To further confirm the observation, we performed SMARCA4 ChIP-PCR in H1299 cells using two distinct primer pairs: one pair specifically targets the SMARCA4-binding region within the Wnt10A promoter while the other targets a SMARCA4-non-binding region upstream of the Wnt10A promoter (serving as the negative control, NC) (Fig. 6g). As a result, Wnt10A promoter amplification was detected in DNA pulled down via SMARCA4 ChIP, but no amplification for the NC fragment. Collectively, these data indicate that SMARCA4 transcriptionally regulates Wnt10A expression via direct binding to the Wnt10A promoter.

**Fig. 7:**
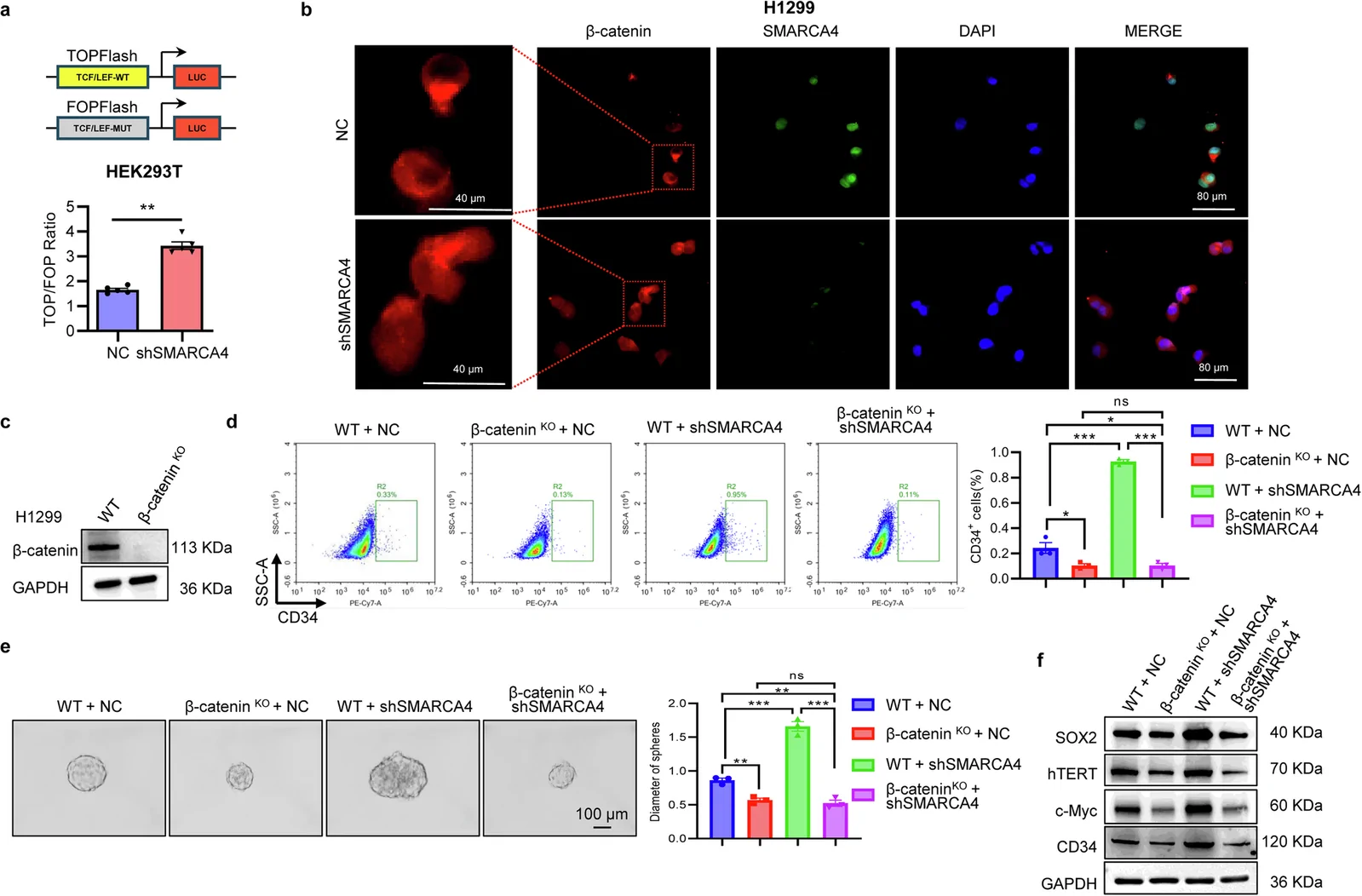
Activation of the Wnt/β-catenin pathway mediated the SMARCA4 deficiency-induced cancer cell stemness. **a** TOP/FOPFlash luciferase assay demonstrating the activation of Wnt signaling in SMARCA4-deficient H1299 cells. **b** Immunofluorescence staining analysis revealing the translocation of β-catenin from the cytoplasm to the nucleus in SMARCA4-deficient H1299 cells. The nucleus was stained with DAPI. Scale bars: 40 and 80 µm. **c** Western blot analysis of β-catenin in WT and β-catenin KO H1299 cells. Cancer cell stemness assessments including flow cytometry analysis of CD34+ cancer stem cells (**d**), tumorsphere formation assay (**e**), and western blot analysis of four stemness marker genes (CD34, hTERT, c-Myc, and SOX2) (**f**) in WT and β-catenin KO H1299 cells with or without expression of SMARCA4. Data are presented as mean ± SEM (*n* = 3). ns: no significance; *P ≤ 0.05; **P ≤ 0.01; ***P ≤ 0.001.

Given the well-established role of Wnt signaling in regulating CSCs, we assessed Wnt signaling activity in H1299 cells with and without SMARCA4 knockdown. The TOP/FOPFlash luciferase assay demonstrated enhanced Wnt signaling activity after SMARCA4 knockdown (Fig. 7a). In addition, we performed immunofluorescence staining to visualize the expression levels and subcellular localization of both SMARCA4 and β-catenin. Notably, we observed the translocation of β-catenin from the cytoplasm to the nucleus in response to SMARCA4 knockdown in H1299 cells (Fig. 7b). To further validate that SMARCA4 deficiency induces cancer cell stemness in H1299 cells *via* the Wnt/β-catenin signaling pathway, we generated β-catenin knockout (β-catenin KO) H1299 cells using the CRISPR-Cas9 technique (Fig. 7c), followed by comprehensive stemness assessments including quantification of CD34+ cell population (Fig. 7d), tumorsphere formation assays (Fig. 7e), and gene expression of those stemness markers including CD34, hTERT, c-Myc, and SOX2 (Fig. 7f). As a result, β-catenin KO in H1299 cells significantly reduced cancer cell stemness, while SMARCA4 knockdown (shSMARCA4) in H1299 cells significantly enriched the cancer stem cell population. Notably, in β-catenin KO H1299 cells, shSMARCA4 did not induce further increases in cancer cell stemness. Taken together, these results demonstrate that SMARCA4 deficiency promotes cancer cell stemness in a Wnt/β-catenin-dependent manner.

### Application of a small molecule of Wnt inhibitor in treatment of SMARCA4-UT

**Fig. 8:**
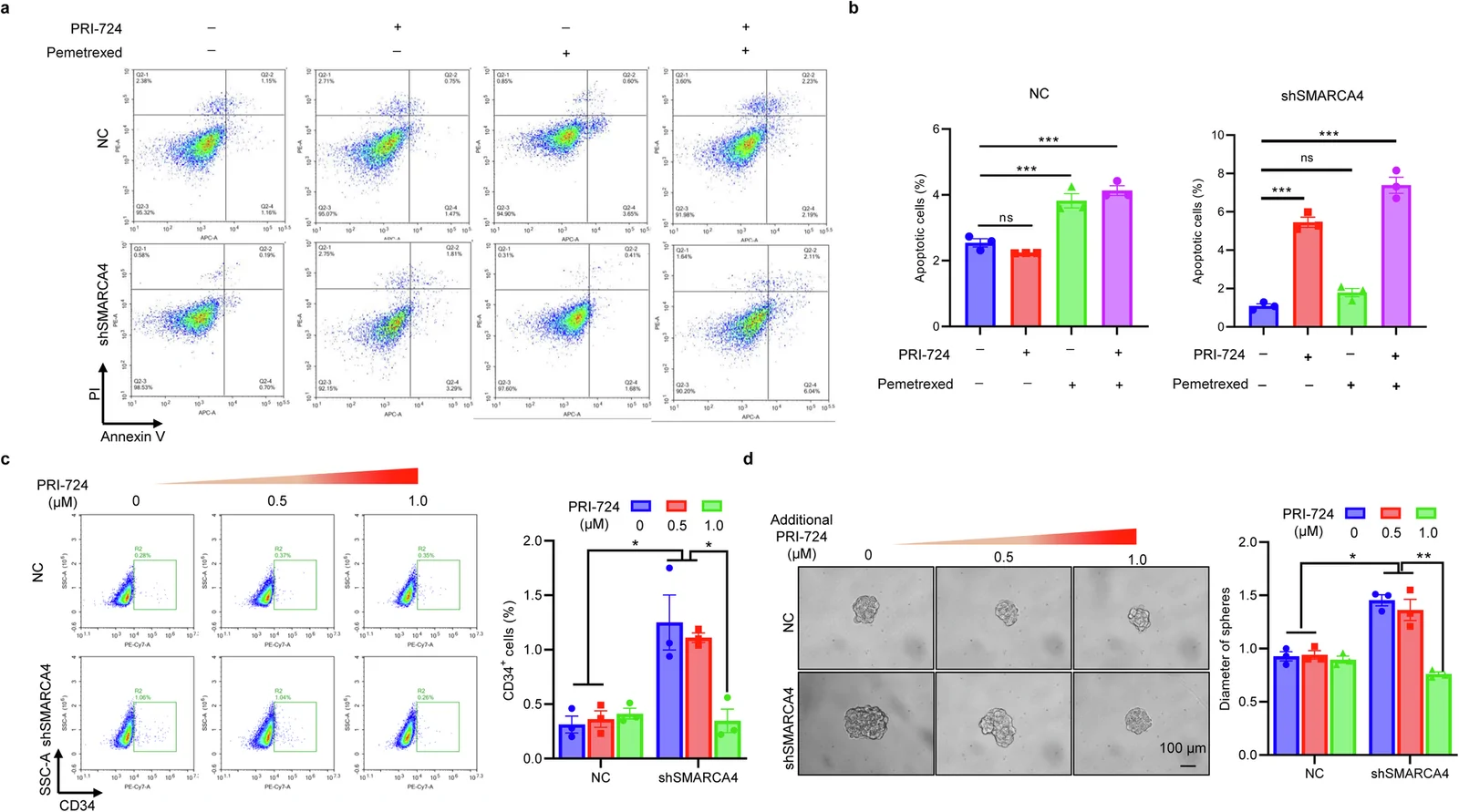
Induction of cellular apoptosis by the Wnt inhibitor PRI-724 in SMARCA4-deficient cancer cells. **a** Annexin V-FITC/PI staining analysis of wild-type (WT) and SMARCA4-deficient H1299 cells treated with PRI-724 and pemetrexed, either individually or in combination. **b** Quantitative analysis of apoptosis (addition of early and late apoptotic cells) in each group in (**a**). Flow cytometry analysis of CD34+ cancer stem cells (**c**) and tumorsphere formation assays (**d**) using NC and shSMARCA4 H1299 cells under treatment with the Wnt inhibitor PRI-724 at three concentrations (0, 0.5, and 1.0 μM). Data are presented as mean ± SEM (*n* = 3). ns: no significance; *P ≤ 0.05; **P ≤ 0.01; ***P ≤ 0.001.

Based on the findings of the current study, we suggest that Wnt signaling pathway-induced cancer cell stemness plays a significant role in regulating thoracic SMARCA4-UT. To explore its potential for clinical application, we utilized PRI-724, a small molecule inhibitor targeting Wnt signaling, to treat WT and SMARCA4-deficient H1299 cells, both alone and in combination with the chemotherapeutic drug Pemetrexed. The Annexin V-FITC/PI staining assay was performed to assess cell apoptosis before and after treatment (Fig. 8a). The combination of PRI-724 and Pemetrexed showed a synergistic effect in the induction of cell apoptosis, while PRI-724 induced apoptosis in SMARCA4-deficient H1299 cells (Fig. 8b).

To further determine whether SMARCA4 regulates the stemness properties of cancer cells *via* the Wnt signaling pathway, we treated H1299 cells with or without SMARCA4 knockdown with the Wnt inhibitor PRI-724 at three concentrations (0, 0.5, and 1.0 μM). Following treatment, cells were analyzed *via* either flow cytometry to quantify CD34+ cancer stem cells or tumorsphere formation assays. As shown in Fig. 8c, SMARCA4 knockdown (shSMARCA4) H1299 cells contained ~3 times more CD34+ cancer stem cells than the control cells (NC). Treatment with 1.0 μM PRI-724 reduced the percentage of CD34+ cancer stem cells in shSMARCA4 H1299 cells to the baseline level similar to that in control. In contrast, the same concentration of PRI-724 had no significant effect on CD34+ cell percentage in control H1299 cells, indicating that the SMARCA4 knockdown-induced cancer cell stemness is dependent on Wnt signaling activity. Consistent with these findings, tumorsphere formation assays yielded comparable results that shSMARCA4 enhanced tumorsphere formation, an effect that was fully reversed by 1.0 μM PRI-724 treatment (Fig. 8d).

**Fig. 9:**
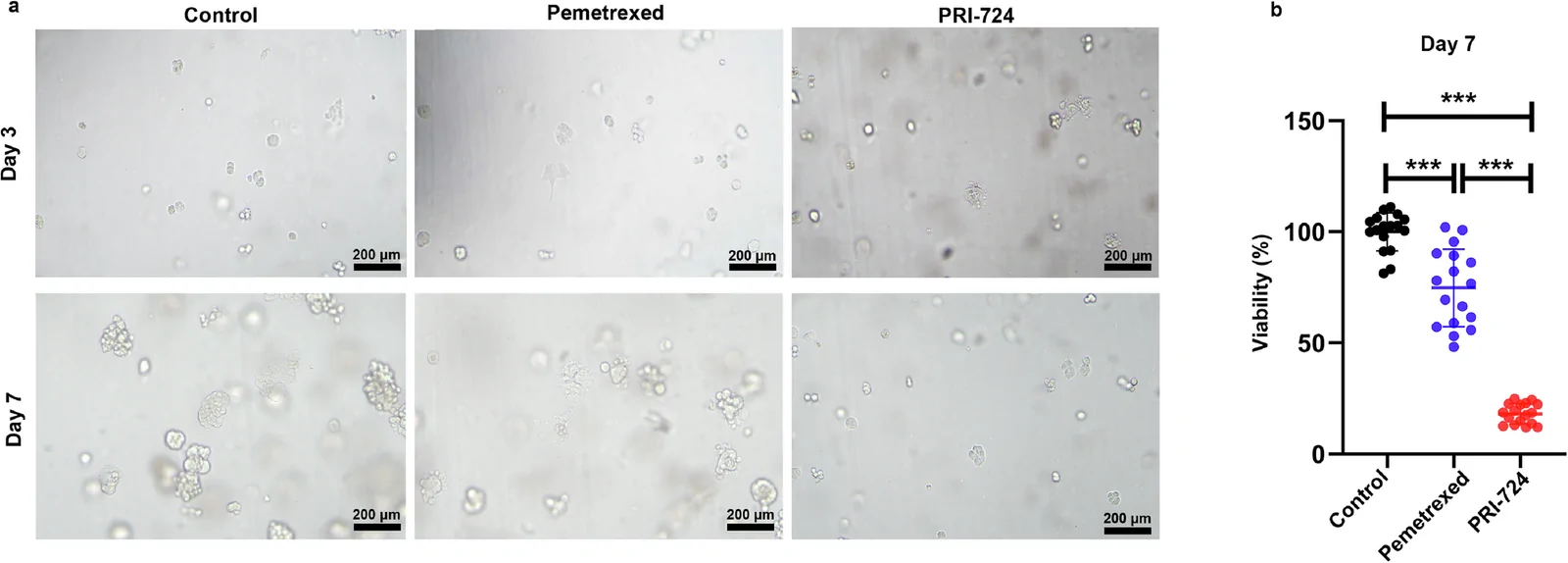
Growth suppression of SMARCA4-UT patient-derived organoids by PRI-724 treatment. **a** Representative images of thoracic SMARCA4-UT patient-derived organoids at day 3 and day 7 after treatment with PRI-724 or pemetrexed, respectively. **b** Quantitative analysis of the vitality of the organoids at day 7 post-treatment as shown in (**a**). ***p ≤ 0.001.

We further evaluated the anti-tumor potential of PRI-724 in thoracic SMARCA4-UT patient-derived organoids, using Pemetrexed as a positive control (Fig. 9a). As shown in Fig. 9b, after 7 days of treatment, PRI-724 significantly inhibited organoid growth compared to Pemetrexed, highlighting the therapeutic potential of PRI-724 in targeting Wnt signaling for the treatment of thoracic SMARCA4-UT.

## Discussion

Thoracic SMARCA4-UT is a pathologically distinct and highly aggressive type of malignant cancer characterized by SMARCA4 deficiency, poor prognosis, strong resistance to conventional chemotherapy, and a high risk of relapse. Identifying the molecular alterations resulting from SMARCA4 deficiency is crucial for understanding the mechanisms underlying SMARCA4-UT pathogenesis and for developing new therapeutic strategies.

In the current study, we applied NSCLC cell lines with shRNA-mediated knockdown of SMARCA4 to mimic thoracic SMARCA4-deficient tumor cells. How to prepare the disease model of thoracic SMARCA4-UT remains a challenge since the origin of the tumors is yet to be determined. Rekhtman et al. reported connections between thoracic SMARCA4-UT and NSCLC by proposing that the former may be developed from SMARCA4‑deficient NSCLC in a stepwise manner through further loss of SMARCA2 [16]. SMARCA4 knockout is embryonically lethal in mice. Glaros S. et al. developed a lung-specific conditional knockout of BRG1 (SMARCA4) using lung-specific promoter Clara cell–secreted protein (CCSP)-Cre, and examined the effect of SMARCA4 deficiency in an ethyl carbamate-induced lung carcinogenesis mouse model [17]. They found that biallelic inactivation of SMARCA4, even combined with SMARCA2 knockout, could not initiate tumor development in untransformed cells, but significantly potentiated cancer development and tumor progression [17, 18]. As such, we used SMARCA4 knockdown NSCLC cells and thoracic SMARCA4-UT patient-derived organoids to reproduce the biology of thoracic SMARCA4-UT.

We demonstrated a connection between SMARCA4 deficiency and CSC properties. Our analysis of patient tissues found that thoracic SMARCA4-UT tumors exhibited significantly higher levels of CSC markers, such as SOX2, CD34, c-Myc, and EpCam, as well as increased level of cell proliferation marker Ki67, compared to adjacent normal tissues. The stable knockdown of SMARCA4 in NSCLC cells resulted in significant promotion of cell proliferation, migration, invasion, and tumorsphere formation.

Our mechanism studies revealed that SMARCA4 deficiency induced the expression of the Wnt ligand *Wnt10A* at the transcriptional level and activated the canonical Wnt/β-catenin signaling pathway. Application of a small molecule inhibitor of the Wnt pathway induced cell apoptosis, inhibited the growth of tumor organoids, and attenuated SMARCA4 knockdown-induced cell stemness, suggesting its potential to be developed as a therapeutic target to treat thoracic SMARCA4-UT.

Currently, there remains a lack of standardized treatment protocols and guidelines for patients with thoracic SMARCA4-UT, particularly those with advanced-stage disease. Conventional chemotherapy remains the most commonly used treatment approach in clinical practice. However, emerging evidence suggests that immune checkpoint inhibitors (ICIs) may be effective for a subset of patients with thoracic SMARCA4-UT, either as monotherapy or in combination with chemotherapy [19–22]. The current study demonstrates that SMARCA4 deficiency induces cancer cell stemness, supporting a novel therapeutic strategy for treating SMARCA4-UT by targeting cancer stem cells. We have validated this approach through preliminary intervention experiments using a Wnt signaling inhibitor. Further in-depth animal studies and clinical trials are needed to confirm the efficacy of this strategy, either when targeting cancer stem cells alone or in combination with chemotherapy and/or ICIs.

## Materials and methods

### Clinical samples

The clinical samples of patients were collected from Shanghai East Hospital. This study was approved by the Institutional Review Board (IRB) of Shanghai East Hospital, Tongji University School of Medicine (approval No. 2025YYS-018). Written consents were obtained from patients providing tissue samples.

### Animal study

6-8-week-old male BALB/c nude mice were purchased from Silaike Animal Company (Shanghai, China). All animal studies were approved by the Institutional Animal Care and Use Committee of Tongji University School of Medicine. 5 × 10^6^ cancer cells were mixed with Matrigel (volume 1:1), followed by subcutaneous injection in the back of each mouse. Mice were randomly assigned to being injected with untreated NCI-H1299 cells or H1299 cells transfected with shSMARCA4 shRNA. Tumor growth was tracked by measuring the volumes of all tumors every 5 days until day 25 after cell transplantation. Tumor volumes were calculated by a formula (length × width²)/2.

### Cell lines and cell culture

NCI-H1299 and NCI-H460 cells were purchased from American Type Culture Collection (ATCC), and maintained in our lab. Cells were cultured in DMEM medium supplemented with 10% Fetal Bovine Serum (Gibco) and 1% penicillin/streptomycin (MACGENE).

### Immunohistochemistry (IHC)

IHC staining was conducted as previously described [23]. Briefly, tumor tissue samples were fixed with 4% paraformaldehyde (Beyotime) and then processed for paraffin embedding and sectioning. Primary antibodies included BRG1/SMARCA4 (ZSGB-BIO, ZA-0673), Ki67 (ZSGB-BIO, ZM-0166), SOX2 (ZSGB-BIO, ZA-0571), CD34 (ZSGB-BIO, ZA-0550), EpCAM (ZSGB-BIO, ZM-0359), and c-Myc (ZSGB-BIO, ZA-0555). Poly-HRP IgG (Leica Biosystems, 83062) was used as the secondary antibody. Images were captured using a 3DHISTECH scanner, and quantitative analysis was performed using CaseViewer.

### SMARCA4 knockdown

Stable knockdown of SMARCA4 was achieved by transduction of a lentiviral-based shRNA vector (Qiachang). SMARCA4-targeting shRNA sequences were as follows: SMARCA4 shRNA1: ACCTTCGAGCAGTGGTTTAAC; SMARCA4 shRNA2: CAAGATGTCGATGATGAATAT; SMARCA4 shRNA3: CTTTGCGTATCGCGGCTTTAA; a negative control (NC) sequence was GGATCCGAGCTCGGTACCAAGCTT. Puromycin (2 μg/mL, ShareBio) was used for positive cell screening. SMARCA4 knockdown efficiency was validated by Western blot and real-time quantitative PCR (RT-qPCR).

### Western blot

Cells were lysed in RIPA buffer containing protease and phosphatase inhibitors. Protein concentration was determined using BCA assay (Beyotime). 30 μg of cell lysates were separated by SDS-PAGE and transferred to PVDF membranes. After blocking with 5% non-fat milk for 1 h, membranes were incubated with primary antibodies (BRG1/SMARCA4, ABclonal, A19556, 1:1000 dilution; SOX2, Cell Signaling Technology, 3579S, 1:1000 dilution; hTERT, Santa Cruz Biotechnology, sc-377511, 1:1000 dilution; c-Myc, Cell Signaling Technology, 9402S, 1:1000 dilution; CD34, Abclonal, A23924PM, 1:1000 dilution; SNAIL1, Santa Cruz Biotechnology, sc-271977, 1:1000 dilution; SLUG, Santa Cruz Biotechnology, sc-166476, 1:1000 dilution; N-Cadherin, Cell Signaling Technology, 13116S, 1:1000 dilution; Wnt10A, Huabio, HA500356, 1:1000 dilution; GAPDH, ABclonal, A19056, 1:10000 dilution) overnight at 4 °C, followed by incubation with HRP-conjugated secondary antibodies (Cell Signaling Technology, 7074S, 1:1000 dilution; Cell Signaling Technology, 7076S, 1:1000 dilution) for 1 h at room temperature. Protein bands were visualized using ECL detection reagents (ShareBio).

### RNA extraction and RT-qPCR

Total RNA was extracted using TRIzol™ reagent (Invitrogen). RNA concentration and purity were determined spectrophotometrically. cDNA was synthesized from 1 μg of total RNA by reverse transcription (Vazyme). RT-qPCR reaction conditions were 95 °C for 10 min, followed by 40 cycles of 95 °C for 15 s and 60 °C for 1 min. GAPDH was used for normalization. All reactions were performed in triplicate with three independent repeats. The primer sequences were: SMARCA4-Forward: GACCAGCACTCCCAAGGTTAC, SMARCA4-Reverse: CTGGCCCGGAAGACATCTG; SOX2-Forward: AATGCCTTCATGGTGTGGT, SOX2-Reverse: CTTCTCCGTCTCCGACAAA; hTERT-Forward: ACAGACCTCCAGCCGTACAT, hTERT-Reverse: GCACATGAAGCGTAGGAAGA; c-Myc-Forward: GGCTCCTGGCAAAAGGTCA, c-Myc-Reverse: CTGCGTAGTTGTGCTGATGT; CD34-Forward: AATCAGCACAGTGTTCACCAC, CD34-Reverse: TGCCCTGAGTCAATTTCACTTC; Snail 1-Forward: TCGGAAGCCTAACTACAGCG, Snail 1-Reverse: AGATGAGCATTGGCAGCGAG; Slug-Forward: CGAACTGGACACACATACAGTG, Slug-Reverse: CTGAGGATCTCTGGTTGTGGT; N-Cadherin-Forward: GCGTCTGTAGAGGCTTCTGG, N-Cadherin-Reverse: GCCACTTGCCACTTTTCCTG; Wnt10A-Forward: GGTCAGCACCCAATGACATTC, Wnt10A-Reverse: TGGATGGCGATCTGGATGC; GAPDH-Forward: TGCACCACCAACTGCTTAGC, GAPDH-Reverse: GGCATGGACTGTGGTCATGAG.

### CCK-8 Assay

Cells were seeded in 96-well plates at a density of 1 × 10³ cells per well. After incubation for 24, 48, and 72 h at 37°C, 10 μL of CCK-8 reagent (ShareBio) was added to each well and incubated for 3 h. OD values were measured at 450 nm using a microplate reader. All experiments were performed with eight replicates and repeated three times independently.

### Colony formation assay

Cancer cells were seeded in 12-well plates for 12 days of incubation at 37 °C with 5% CO₂ with the medium changed every 3 days. Eventually, the colonies were fixed with 4% paraformaldehyde and stained with 0.5% crystal violet.

### Tumorsphere formation assay

Cancer cells were suspended in serum-free DMEM/F12 medium supplemented with 2% B27 (Gibco), 20 ng/mL human recombinant epidermal growth factor (MACGENE), 20 ng/mL human recombinant basic fibroblast growth factor (MACGENE), and 1% penicillin-streptomycin, followed by incubation at 37 °C and 5% CO₂ for 10 days. The diameter of each tumorsphere was measured under microscope.

### Wound healing assay and transwell assay

For wound healing cell migration assay, cells were cultured to 90-95% confluence, a sterile 200 μL pipette tip was used to create a straight scratch in the cell monolayer. Detached cells were removed by PBS-washing, and then cultured in 1% FBS medium. Wound closure was quantified using ImageJ software. For transwell cell invasion assay, upper chambers were pre-coated with Matrigel (1 mg/mL, Corning), and seeded with 5,000 cells in each well in DMEM/F12 medium. Regular medium with 10% FBS was used in bottom chambers. After 12 h incubation, invaded cells were fixed with 4% PFA, stained with 0.1% crystal violet.

### Immunofluorescence staining

Cells attached on glass slips were fixed, permeabilized, and blocked, followed by incubation with primary antibodies: BRG1/SMARCA4 (ABclonal, A19556,1:500 dilution), β-Catenin (Cell Signaling Technology, 8480S,1:400 dilution) overnight at 4 °C. Fluorescent secondary antibodies (Alexa Fluor 488 and 555, respectively) were used to incubate for 1 h at room temperature. Nuclei were stained by DAPI.

### TOP/FOPflash assay

Cells were co-transfected with TOPflash (TCF/LEF reporter) or FOPflash (mutant control) plasmid and pRL-TK luciferase plasmid. After 48 h, luciferase activity was measured using the Dual-Luciferase Reporter Assay System. Firefly luciferase activity was normalized to Renilla luciferase activity. TOP/FOPflash ratio was calculated to determine TCF/LEF transcriptional activity.

### Chromatin immunoprecipitation (ChIP)-PCR assay

The ChIP-PCR kit was purchased from Beyotime Biotechnology (#P2080S). Cells were cross-linked with 1% formaldehyde at room temperature for 10 min, and terminated by adding 125 mM glycine. After being washed twice with ice-cold PBS, cells were harvested and lysed in ChIP lysis buffer supplemented with protease inhibitors. Subsequent chromatin DNA sonication, incubation with protein A/G agarose beads and anti-BRG1/SMARCA4 (Abclonal, #A19556) or normal IgG, SMARCA4-binding DNA elution and purification were performed following the manufacturer’s instructions. The isolated DNA was then subjected to PCR analysis. The primers for Wnt10A binding region are: Forward: 5’ CTTACCCTTGAGAGGCACCG 3’, Reverse: 5’ CGACACACAGCTCCGACTC 3’. The primers for negative control (NC) are: Forward: 5’ GGGACAGGAAGATCTCAGCG 3’, Reverse: 5’ TTAGCGGAAGAGCGAGAACC 3’.

### Flow cytometry analysis of CD34+ cells

Approximately 1 × 10^6^ cells were incubated with fluorochrome-conjugated anti-CD34 (eBioscience™, #25-0349-42) or the corresponding IgG control for 30 min at 4 °C in the dark. Then cells were washed twice, resuspended in PBS for flow cytometric analysis. Dead cells and debris were excluded based on forward and side scatter parameters. The percentage of CD34-positive cells was determined by flow cytometry and FlowJo software. All experiments were conducted in triplicate.

### β-catenin knockout (KO) using CRISPR-Cas9 technique

The gβ-catenin-Cas9 vector was constructed by cloning a specific single guide RNA (sgRNA) sequence targeting β-catenin (5’-TACCCAGCGCCGTACGTCCA-3’) into the lentiCRISPRv2 vector (Addgene plasmid #98290) at the BsmBI restriction site.

### Apoptosis assay

Annexin V and propidium iodide (PI) staining (ShareBio) was used to analyze cell apoptosis using a flow cytometer according to manufacturer’s instructions. Early and late apoptotic cells were calculated. All experiments were repeated for three times independently.

### Organoids

SMARCA4-UT patient-derived organoids were purchased from ACCURATE International Biotechnology (Guangzhou) Co., Ltd. They were treated with PRI-724 and pemetrexed, either individually or in combination. Cell viability was measured by ATP-tumor chemosensitivity assay (ATP-TCA) using the Accuroid Reagent (Accurate International) according to manufacturer’s instructions.

### RNA-seq analysis

Briefly, RNA-seq libraries were constructed using VAHTS Universal V10 RNA-seq Library Prep Kit (Vazyme) according to manufacturer’s instructions and sequenced on an Illumina Novaseq X Plus platform. Raw reads of fastq format were processed using the fastp software, and the low-quality reads were removed. Clean reads were mapped to the reference genome using HISAT2. Fragments Per Kilobase of transcript per Million mapped reads (FPKM) of each gene was calculated, and the read counts of each gene were obtained by HTSeq-count. The expressed genes were filter by FPKM. Differentially expressed genes were analyzed by using DESeq2. Q value < 0.05 and fold change ≥ 2 or fold change ≤ 0.5 was set as the threshold for significantly differentially expressed genes (DEGs). Hierarchical cluster analysis and Wikipathway enrichment analysis of DEGs were performed using R (V3.2.0).

### Gene set enrichment analysis (GSEA)

GSEA was conducted using the GSEA software to identify and visualize pathways differentially regulated between the shSMARCA4 and NC groups. Predefined gene sets were obtained from Gene Ontology (GO), Kyoto Encyclopedia of Genes and Genomes (KEGG), and WikiPathway, respectively. Significantly enriched gene sets (*p* ≤ 0.05) were ranked according to their normalized enrichment score (NES).

### Statistical analysis

Data are presented as mean ± SEM unless otherwise stated. The two-tailed Student’s *t* test was used in statistical comparisons. *p* ≤ 0.05 was considered significant. Experiments were repeated at least 3 times unless otherwise stated.

## Supplementary information

original data

## Data Availability

All data are included in this article. The RNA-seq data has been deposited at the China National Center for Bioinformation (PRJCA063474). All materials generated by ourselves are shareable upon request.
